# Biomarkers from circulating neutrophil transcriptomes have potential to detect unruptured intracranial aneurysms

**DOI:** 10.1186/s12967-018-1749-3

**Published:** 2018-12-28

**Authors:** Vincent M. Tutino, Kerry E. Poppenberg, Lu Li, Hussain Shallwani, Kaiyu Jiang, James N. Jarvis, Yijun Sun, Kenneth V. Snyder, Elad I. Levy, Adnan H. Siddiqui, John Kolega, Hui Meng

**Affiliations:** 1 Canon Stroke and Vascular Research Center, University at Buffalo, Clinical and Translational Research Center, 875 Ellicott Street, Buffalo, NY 14214 USA; 2 Department of Biomedical Engineering, University at Buffalo, Buffalo, NY USA; 3 Department of Computer Science and Engineering, University at Buffalo, Buffalo, NY USA; 4 Department of Neurosurgery, Jacobs School of Medicine and Biomedical Sciences, University at Buffalo, Buffalo, NY USA; 5 Genetics, Genomics, and Bioinformatics Program, University at Buffalo, Buffalo, NY USA; 6 Department of Pediatrics, Jacobs School of Medicine and Biomedical Sciences, University at Buffalo, Buffalo, NY USA; 7 Department of Microbiology and Immunology, University at Buffalo, Buffalo, NY USA; 8 Department of Radiology, Jacobs School of Medicine and Biomedical Sciences, University at Buffalo, Buffalo, NY USA; 9 Department of Neurology, Jacobs School of Medicine and Biomedical Sciences, University at Buffalo, Buffalo, NY USA; 10 Department of Pathology and Anatomical Sciences, Jacobs School of Medicine and Biomedical Sciences, University at Buffalo, Buffalo, NY USA; 11 Department of Mechanical & Aerospace Engineering, University at Buffalo, Buffalo, NY USA

**Keywords:** Intracranial aneurysm, Machine learning, Transcriptomics, Neutrophils, Inflammation

## Abstract

**Background:**

Intracranial aneurysms (IAs) are dangerous because of their potential to rupture and cause deadly subarachnoid hemorrhages. Previously, we found significant RNA expression differences in circulating neutrophils between patients with unruptured IAs and aneurysm-free controls. Searching for circulating biomarkers for unruptured IAs, we tested the feasibility of developing classification algorithms that use neutrophil RNA expression levels from blood samples to predict the presence of an IA.

**Methods:**

Neutrophil RNA extracted from blood samples from 40 patients (20 with angiography-confirmed unruptured IA, 20 angiography-confirmed IA-free controls) was subjected to next-generation RNA sequencing to obtain neutrophil transcriptomes. In a randomly-selected training cohort of 30 of the 40 samples (15 with IA, 15 controls), we performed differential expression analysis. Significantly differentially expressed transcripts (false discovery rate < 0.05, fold change ≥ 1.5) were used to construct prediction models for IA using four well-known supervised machine-learning approaches (diagonal linear discriminant analysis, cosine nearest neighbors, nearest shrunken centroids, and support vector machines). These models were tested in a testing cohort of the remaining 10 neutrophil samples from the 40 patients (5 with IA, 5 controls), and model performance was assessed by receiver-operating-characteristic (ROC) curves. Real-time quantitative polymerase chain reaction (PCR) was used to corroborate expression differences of a subset of model transcripts in neutrophil samples from a new, separate validation cohort of 10 patients (5 with IA, 5 controls).

**Results:**

The training cohort yielded 26 highly significantly differentially expressed neutrophil transcripts. Models using these transcripts identified IA patients in the testing cohort with accuracy ranging from 0.60 to 0.90. The best performing model was the diagonal linear discriminant analysis classifier (area under the ROC curve = 0.80 and accuracy = 0.90). Six of seven differentially expressed genes we tested were confirmed by quantitative PCR using isolated neutrophils from the separate validation cohort.

**Conclusions:**

Our findings demonstrate the potential of machine-learning methods to classify IA cases and create predictive models for unruptured IAs using circulating neutrophil transcriptome data. Future studies are needed to replicate these findings in larger cohorts.

**Electronic supplementary material:**

The online version of this article (10.1186/s12967-018-1749-3) contains supplementary material, which is available to authorized users.

## Background

Intracranial aneurysm (IA) rupture is the primary cause of nontraumatic subarachnoid hemorrhage and its devastating sequalae [1]. The risk of rupture can be reduced by elective endovascular or surgical treatment [2, 3]. However, because IAs are almost invariably asymptomatic until rupture [4, 5], unruptured aneurysms are usually detected incidentally in individuals who are prescribed cerebral imaging for other reasons. A blood test capable of identifying individuals who harbor unruptured aneurysms would therefore be a significant advance in the field.

Circulating blood is a dynamic, information-rich tissue that continuously interacts with the aneurysm. In many different disease states, transcriptome profiling has been used to discover panels of differentially expressed genes in the circulating blood that could serve as useful diagnostic markers [6]. This strategy has been successfully applied to complex vascular diseases, including IA [7–9]. In a recent case–controlled study, we performed transcriptome profiling on circulating neutrophils from patients with and without IA and discovered an IA-associated RNA expression signature (NCBI Gene Expression Omnibus Accession Number GSE106520) [10]. This signature was characteristic of peripheral neutrophil activation and was able to separate patients with IA from controls in several statistical analyses. Although these results showed that differences in neutrophil expression may be able to distinguish patients with IA, biomarker development from expression data is more complex than the identification of differentially expressed genes. Average expression differences between patients with and without IA cannot themselves be used to predict aneurysm on an individual basis [11]. Rather, classification algorithms that use discrete expression levels of informative transcripts to predict the presence of IA from individual samples are required.

In this study, we sought to test the feasibility of creating classification models of unruptured IAs based on RNA expression of circulating neutrophils. We recruited additional patients with and without unruptured IAs (confirmed on angiography), isolated peripheral blood neutrophils, and performed next-generation RNA sequencing to obtain the neutrophil transcriptomes. Differential expression analysis was used to identify highly significantly differentially expressed transcripts as features for model development. As there was no precedent for the specific type of algorithm best suited for IA detection from neutrophil expression differences, we applied four widely used supervised machine-learning approaches to select a classification model most fitting for our data. Trained classification models were then validated in an independent testing dataset of neutrophil transcriptomes. Furthermore, real-time quantitative polymerase chain reaction (qPCR) was used to corroborate expression differences of model transcripts in neutrophil samples from a new, separate validation cohort of patients with and without IA. Results from this study could lay the groundwork for future, larger efforts towards developing a blood-based IA diagnostic.

## Methods

### Study population

This study was approved by the University at Buffalo Health Sciences Institutional Review Board (Study No. 030-474433). Methods were carried out in accordance with the approved protocol and written informed consent was obtained from all subjects. We included individuals who were older than 18 years, spoke English, and had not received previous treatment for IA. We excluded individuals with potentially altered immune systems; including patients who were pregnant, had recently undergone invasive surgery, were undergoing chemotherapy, had a body temperature above 37.78 °C (100 °F), had received solid organ transplants, had autoimmune diseases, and those who were taking prednisone or any other immunomodulating drugs.

Between December 2013 to May 2016, 106 peripheral blood samples were collected from patients undergoing cerebral digital subtraction angiography (DSA) at Gates Vascular Institute in Buffalo, New York: 51 patients had a positive IA diagnosis and 55 had a negative IA diagnosis (controls). DSA imaging was used to confirm IA diagnosis by either positive or negative angiographic presence of IA. Patient medical electronic records were also collected. Reasons for the patients to receive DSA included confirmation of findings from noninvasive imaging of the presence of unruptured IAs, vascular malformations, or carotid stenosis, or follow-up noninvasive imaging of previously detected IAs. Prior to RNA expression analysis, we further excluded patients with other known cerebrovascular malformations or extracranial aneurysms, including abdominal aortic aneurysms. The presence of other cerebrovascular malformations or extracranial aneurysms was recorded from both the patient’s operative report following DSA and their recorded medical history.

### Neutrophil isolation

During the DSA procedure, 16 mL of blood was drawn from the access catheter in the femoral artery and transferred into two 8 mL, citrated, cell preparation tubes (BD, Franklin Lakes, NJ). Neutrophils were isolated within 1 h of peripheral blood collection, as described elsewhere [12]. Cell preparation tubes were centrifuged at 1700×*g* for 25 min to separate erythrocytes and neutrophils from mononuclear cells and plasma in the peripheral blood samples via a Ficoll density gradient. Erythrocytes and neutrophils were collected into a 3 mL syringe. Following hypotonic lysis of red blood cells, neutrophils were isolated by centrifugation at 400×*g* for 10 min and disrupted and stored in TRIzol reagent (Life Technologies, Carlsbad, CA) at − 80 °C until further processing. Neutrophils isolated in this fashion are more than 98% CD66b+ by flow cytometry and contain no contaminating CD14+ monocytes [13].

### RNA preparation

Neutrophil RNA was extracted using TRIzol, according to the manufacturer’s instructions. Trace DNA was removed by DNase I (Life Technologies, Carlsbad, CA) treatment. RNA was purified using the RNeasy MinElute Cleanup Kit (Qiagen, Venlo, Limburg, Netherlands) and suspended in RNase-free water. The purity and concentration of RNA in each sample were measured by absorbance at 260 nm on a NanoDrop 2000 spectrophotometer (Thermo Scientific, Waltham, MA), and 200–400 ng of RNA was sent to our university’s Next-Generation Sequencing and Expression Analysis Core facility for further quality control. Precise RNA concentration was measured at the core facility via the Quant-iT RiboGreen Assay (Invitrogen, Carlsbad, CA) with a TBS-380 Fluorometer (Promega, Madison, WI). The quality of the RNA samples was measured with an Agilent 2100 BioAnalyzer RNA 6000 Pico Chip (Agilent, Las Vegas, NV). RNA samples of acceptable purity (260/280 ratio of ≥ 1.9) and integrity (RIN ≥ 5.0) were considered for RNA sequencing.

### RNA sequencing

RNA libraries were constructed using the Illumina TruSeq RNA Library Preparation Kit (Illumina, San Diego, CA). All samples were subjected to 50-cycle, single-read sequencing in a HiSeq 2500 system (Illumina) and demultiplexed using Bcl2Fastq v2.17.1.14 (Illumina). Gene expression analysis was carried out using the Tuxedo Suite [14–17]. For each sample, short RNA fragment data in FASTQ format was compiled and aligned to the human reference genome (human genome 19—hg19) using TopHat v2.1.13 [17]. To evaluate the quality of RNA sequencing, we performed quality control analysis using FASTQC [18] and visualized and compared the aggregate quality control data using MultiQC [19].

Transcript expression levels were calculated from counts using transcripts per million (TPM) normalization for comparison of RNA levels between samples. Since samples were processed in two batches, we performed batch effect correction using ComBat under the default settings in R [20, 21]. This was performed on expression data for all transcripts with an average TPM > 1.0 in at least one of the two groups (see Additional file 1: Table S1 for batch information).

### Differential expression analysis

Prior to differential expression analysis, neutrophil transcriptomes were randomly divided into two cohorts. Based on standard convention [22, 23], and to maximize learning while retaining a substantial testing group, 3/4 (75%) of the samples were allocated to a training cohort and 1/4 (25%) was allocated to a testing cohort, each containing half IA and half control samples. Although the investigators could not be blinded to sample class, randomization was performed to unbiasedly allocate samples to the training and testing cohorts. Differential gene expression analysis in the training cohort was carried out using F statistics to assess differential variation in the mean on a transcript-by-transcript basis [24–27]. Multiple testing correction was performed by using the John Storey method [28], and q-values were reported for each transcript. Transcripts were considered significantly differentially expressed at an FDR-adjusted p-value (q-value) < 0.05.

### Bioinformatics

We performed gene ontology (GO) term enrichment analysis using the open source Gene Ontology enRIchment anaLysis and visuaLizAtion tool (GORILLA) on all differentially expressed transcripts (q < 0.05) [29]. This was done using a background gene list of previously published neutrophil RNA expression patterns (average fragments per kilo base of transcript per million mapped reads, FPKM > 1.0) of three healthy individuals, described elsewhere [12]. This tool identified GO terms that are enriched in genes with increased or decreased expression in IA compared to the background neutrophil expression using standard hypergeometric statistics. We reported associated GO processes and functions if the enrichment FDR-adjusted p-value (q-value) was < 0.20 (20% FDR).

### Feature selection for classification model development

Prior to model training, the set of differentially expressed transcripts was reduced by filtering. We retained only transcripts with an FDR < 0.05 and absolute fold-change ≥ 1.5. To visualize how those transcripts separated samples from patients with and without IAs, we performed principal component analysis (PCA) in R using the prcomp package under the default settings [30]. We also performed post hoc power estimation following a method by Hart et al. [31] for 15 samples in each group, with an α = 0.05, and a coefficient of variation and counts per million mapped reads of 0.40 and 38, respectively [10].

### Model training

Using the selected transcripts, we trained classification models using MATLAB Statistics and Machine Learning Toolbox (MathWorks, Natick, MA) and R bioconductor (https://www.bioconductor.org/). Specifically, we used four algorithms that have been successfully used for disease classification from gene expression data [32]. These machine-learning methods included cosine nearest neighbors (cosine NN) classification [33], diagonal linear discriminant analysis (DLDA) [34], nearest shrunken centroids (NSC) classification [35], and support vector machines (SVM) [36]. Each method was applied to the training cohort separately and evaluated with a leave-one-out (LOO) cross-validation to estimate model performance and prevent overfitting.

#### k-NN classification

The k-nearest neighbor method [37] with a cosine metric (cosine NN) was employed. The number of neighbors, k, was set as 5 for cosine NN. The resulting model classified test samples by calculating their distance to each training sample. The test sample labels were predicted by choosing the class that was most common among their k-nearest neighbors.

#### LDA

We trained a classifier using diagonal LDA (DLDA), as described elsewhere [33]. This method seeks the linear combination of transcripts that best separates two classes using a diagonal covariance matrix. The linear model coefficients associated with transcripts (discriminant scores) relayed the importance of each transcript to the prediction model [38]. Classification was performed by projecting a test sample onto the maximally separating direction that was determined by discriminant scores and calculating the corresponding posterior probability of IA.

#### Nearest centroids classification

We used a modification of the nearest centroids technique, called NSC [35]. This method calculates class-specific centroids (standard deviation normalized averages) for each transcript and refines them by eliminating those with variable expression. Classification was performed by comparing the expression of the included model transcripts with the centroids of the two classes and assigning it to the class that it was closest to in squared distance [35].

#### SVM

The most complex classification algorithm we implemented was SVM [39]. To separate the binary labeled training samples, SVM finds a hyperplane that is maximally distant from samples of either class. A linear kernel was used in model creation. The resulting model classified test samples by mapping them to a higher-dimensional space and making decisions based on their signed distance to the hyperplane.

### Model assessment in the training cohort

The performance of each model in the training cohort was estimated using the results of the LOO cross-validation. The model classifications were compared to each patient’s clinical diagnosis from imaging, and the true positives (TP), true negatives (TN), false positives (FP), and false negatives (FN) were counted. Each model’s performance was first assessed by calculating the model’s sensitivity, specificity, and accuracy, as follows:

$$Sensitivity = TP/\left( {TP + FN} \right)$$

$$Specificity = TN/\left( {TN + FP} \right)$$

$$Accuracy = \left( {TP + TN} \right)/\left( {TP + FP + FN + TN} \right)$$

Based on model predictions, we created receiver operating characteristic (ROC) curves and calculated the area under the ROC curve (AUC) to assess model performance [40].

### Validation of the models in an independent testing cohort

Classification models were independently tested on transcriptomes from the testing cohort. TPM values of these model features were input into the models for classification of IA presence. The classification results were compared to clinical diagnoses to calculate the true sensitivity, specificity, and accuracy for each model. ROC curves were constructed and AUCs were used to assess the performance of each classifier [41].

### Cross-validation over all samples

Because the models were fit using data points from a randomly selected training dataset (n = 30), selection bias may introduce inconsistency in model predictions. To increase the prediction reliability of the models and to create algorithms more generalizable to a broader population, we implemented LOO cross-validation using the expression levels of the 26 selected transcripts from all 40 patients for each model. The LOO cross-validation method essentially retrained the models in 40 different training sets consisting of 39 samples and performed testing on the remaining sample. As before, classification results were used to calculate sensitivity, specificity, and accuracy for each model, as well as to find the AUC of the ROC curve for each modified classifier.

### Positive and negative predictive values of the models

On the basis of the cross-validation results over all samples, we assessed the predictive value of the classification models by calculating their positive predictive values (PPV) and negative predictive values (NPV) [42]. PPVs and NPVs were estimated using the following formulas based on Bayes’ theorem [43, 44]:

$$PPV = \frac{Sensitivity \times Prevalence }{{Sensitivity \times Prevalence + \left( {1 - Specificity} \right) \times \left( {1 - Prevalence} \right)}}$$

$$NPV = \frac{{Specificity \times \left( {1 - Prevalence} \right) }}{{\left( {1 - Sensitivity} \right) \times Prevalence + Specificity \times \left( {1 - Prevalence} \right)}}$$

The PPV and NPV were calculated over a range of prevalence from 0 to 100%, noting the reported range of IA prevalence (3.2–7%) from the literature [45–48].

### Validation of expression differences by qPCR in an independent validation cohort

To validate expression differences in the 26 genes in our models, quantitative polymerase chain reaction (qPCR) was performed. Due to limitations in mRNA volume, qPCR was performed on seven model transcripts in 10 additional patients (independent cohort of 5 with IA and 5 controls), as described previously [10]. In brief, oligonucleotide primers were designed with a 60 °C melting temperature and a length of 15–25 nucleotides to produce PCR products with lengths of 50–250 base pairs using Primer3 software [49] and Primer BLAST (NCBI, Bethesda, MD). The replication efficiency of each primer set was tested by performing qPCR on serial dilutions of cDNA samples (primer sequences, annealing temperatures, efficiencies, and product lengths are shown in Additional file 1: Table S2).

For reverse transcription, first-strand cDNA was generated from total RNA using OmniScript Reverse Transcriptase kit (Qiagen, Venlo, Limburg, Netherlands) according to the manufacturer’s directions. qPCR was run with 10 ng of cDNA in 25 µL reactions in triplicate in Bio-Rad CFX Connect (Bio-Rad, Hercules, California) using ABI SYBR Green I Master Mix (Applied Biosystems, Foster City, California) and gene-specific primers at a concentration of 0.02 μM each. The temperature profile consisted of an initial step of 95 °C for 10 min, followed by 40 cycles of 95 °C for 15 s and 60 °C for 1 min, and then a final melting curve analysis from 60 °C to 95 °C over 20 min.

Gene-specific amplification was demonstrated by a single peak using the Bio-Rad dissociation melt curve. Samples were normalized based on *GAPDH, 18s rRNA*, and *GPI* expression, which were run in parallel reactions to the genes of interest. These values were used to calculate average fold-change between the two groups using the 2^−ΔΔCt^ method [50]. These values were calculated for each housekeeping gene and averaged. Average fold-change in gene expression measured by qPCR data in the new cohort was then compared to the fold-change calculated from RNA sequencing in the training cohort.

### Influence of IA size on the 26 classifier transcripts

To determine if aneurysm size could affect classification results, we dichotomized the entire IA cohort into a “small” (< 5 mm) group and a “large” (≥ 5 mm) group and analyzed expression differences of the 26 classifier transcripts in samples from patients with IA in the two groups separately. The 5 mm aneurysm size cutoff was based on data reported by the PHASES study [51], which pooled an analysis of six longitudinal investigations [3, 52–56] and found that aneurysms < 5 mm were less likely to rupture than those with larger diameters; IAs between 7 and 10 mm had 2.7 times greater risk of rupture than small IAs (< 5 mm); and IAs > 20 mm had 14.3 times greater risk [51]. We investigated fold-changes in the 26 genes between the “small” IA group and the entire control group (n = 20) and between the “large” IA group and the entire control group (n = 20) to determine if aneurysm size affected their expression pattern.

## Results

### Study participants

During the study period, we collected 106 blood samples (51 from patients with IA, 55 from control subjects) as well as angiographic images and medical records data from individuals undergoing cerebral DSA. Of the blood samples collected, 43 (20 from IA patients, 23 from controls) met our criteria and also had neutrophil RNA of sufficient quality and volume for sequencing. A total of 40 patients (20 with IA and 20 controls) were then chosen and randomly divided into a 30-patient training cohort (n = 15 IA and n = 15 control) and a 10-patient testing cohort (n = 5 IA and n = 5 control). Characteristics of the two cohorts are provided in Table 1. These samples were of sufficient quality and had an average 260/280 ratio of 2.02 (range 1.90–2.12) and an average RNA integrity number (RIN) of 6.88 (range 5.2–8.2) (Additional file 1: Table S3). Patients with IAs had only saccular aneurysms that ranged in size (greatest diameter) from 1 to 19 mm. Five patients with IA had multiple aneurysms (Additional file 1: Table S4). A portion of these samples (n = 22) had been previously analyzed in our aforementioned study that investigated neutrophil expression differences between patients with and without IA [10].

**Table 1:** Clinical characteristics

	Training cohort		Testing cohort	
	Control (n = 15)	Aneurysm (n = 15)	Control (n = 5)	Aneurysm (n = 5)
Age (mean ± SE)	59 ± 4.8	63 ± 2.8	63 ± 7.2	52.6 ± 6.6
Age [median (Q1/Q3)]	61 (52.5/71.5)	64 (56.5/68.5)	68 (62/71)	53 (47/54)
Sex (number of patients)				
Female	40%	66.67%	60%	40%
Smoker (number of patients)				
Yes	0%	20%	40%	60%
Comorbidities (number of patients)				
Hypertension	60%	60%	60%	20%
Heart disease	6.67%	26.67%	40%	0%
High cholesterol	26.67%	40%	60%	0%
Stroke history	6.67%	0%	0%	0%
Diabetes	33.33%	20%	20%	0%
Osteoarthritis	20%	33.33%	20%	0%

Clinical characteristics of the randomly-created training and testing cohorts. With the exception of age, these factors were quantified as binary data points. The clinical factors were retrieved from the patients’ medical records via the latest “Patient Medical History” form administered prior to imaging

### Differential RNA expression in neutrophils from patients with IA vs. controls

**Fig. 1:**
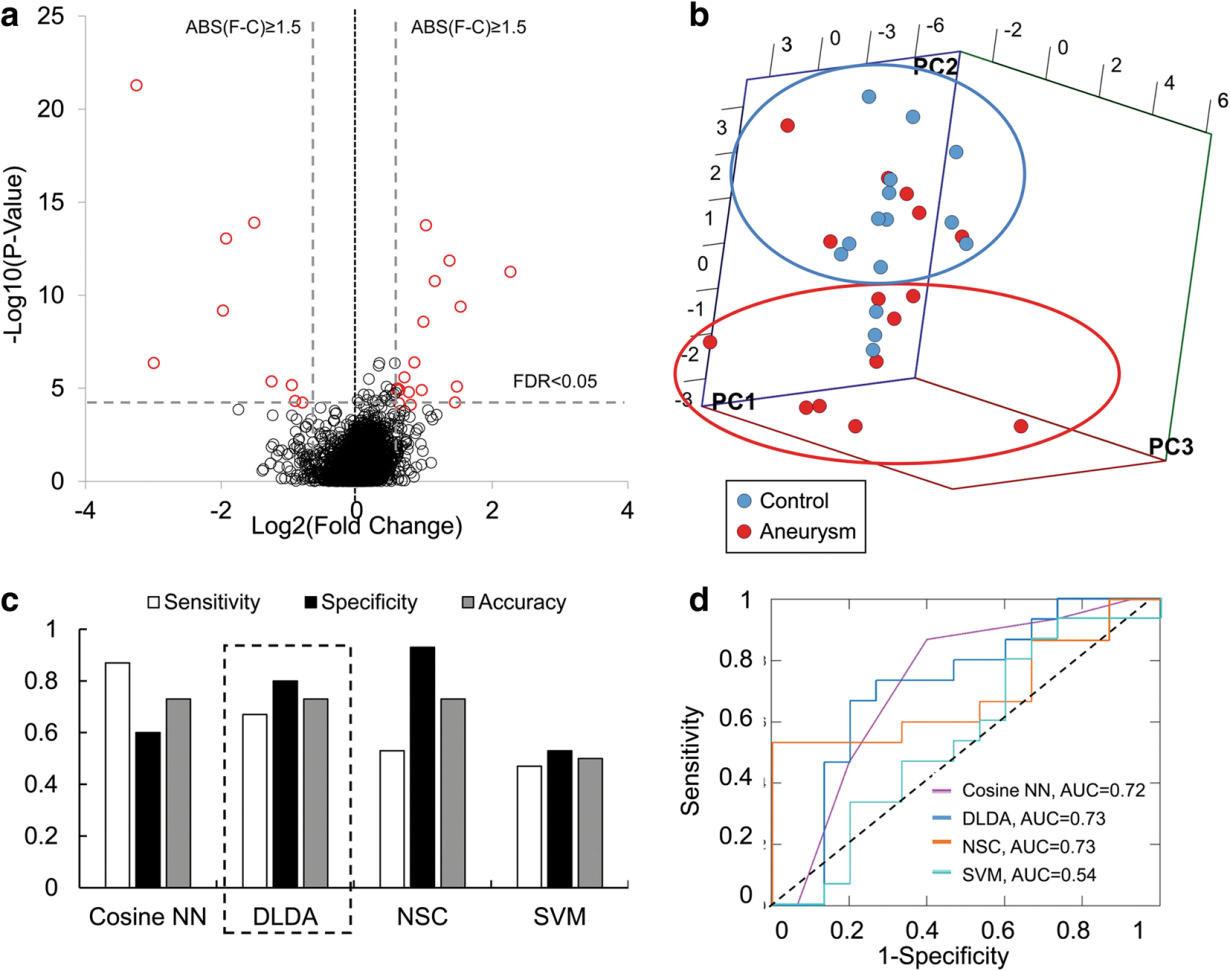
Neutrophil RNA expression differences between patients with intracranial aneurysms (IA) and IA-free controls, feature selection for classification model creation, and model training. **a** Transcriptome profiling demonstrated 95 differently expressed transcripts (q-value < 0.05) between patients with IA and controls. Of these, 26 had a false discovery rate (FDR) < 0.05 and an absolute fold change ≥ 1.5 (in red). **b** Principal component analysis (PCA) using these 26 transcripts demonstrated general separation between samples from patients with IA (60%, circled in red) and those from controls (80%, circled in blue). **c** Estimation of model performance during leave-one-out (LOO) cross-validation in the training cohort demonstrated that most models performed with an accuracy of 0.50–0.73. Among the classification models, diagonal linear discriminant analysis (DLDA) had the highest combination of sensitivity, specificity, and accuracy (0.67, 0.80, 0.73 respectively). **d** Receiver operating characteristic (ROC) analysis using classifications in the training dataset showed that the models had areas under the curve of 0.54 (support vector machines [SVM]) to 0.73 (DLDA). (F-C: fold-change; ABS: absolute value; Cosine NN: cosine nearest neighbors; NSC: nearest shrunken centroids)

RNA sequencing data were used to identify differentially expressed neutrophil transcripts between the 15 patients with IA and 15 controls in the training cohort. Overall, our sequencing experiments had an average of 53.84 million sequences per sample and a 95.4% read mapping rate (% aligned) (Additional file 1: Table S5). The volcano plot in Fig. 1a shows neutrophil expression differences between IA patients and controls in terms of average fold-change in expression and significance level. From 12,775 different transcripts with average TPM > 1.0 across samples in either group, differential expression analysis identified 95 transcripts that were significantly differentially expressed (q < 0.05). Gene set enrichment analysis performed using these 95 differentially expressed transcripts showed that genes with higher levels in the IA group were involved in defense response, leukocyte activation, stem cell maintenance, maintenance of cell number, cell activation, and stem cell development (Table 2). Genes with lower levels in IAs were involved in regulation of glutathione and tetrapyrrole binding.

**Table 2:** Gene ontology (GO) analysis

Category	GO term	Description	p-value	q-value
Transcripts with higher expression in intracranial aneurysms (IA)				
Process	GO:0031347	Regulation of defense response	5.11E−06	0.0658
Process	GO:0050727	Regulation of inflammatory response	1.01E−05	0.0652
Process	GO:0019934	cGMP-mediated signaling	3.77E−05	0.162
Process	GO:0032101	Regulation of response to external stimulus	3.90E−05	0.125
Process	GO:0031348	Negative regulation of defense response	4.45E−05	0.115
Process	GO:0050728	Negative regulation of inflammatory response	5.21E−05	0.112
Process	GO:0007165	Signal transduction	6.64E−05	0.122
Function	GO:0004908	Interleukin-1 receptor activity	2.25E−06	0.00858
Function	GO:0004872	Receptor activity	7.22E−05	0.138
Function	GO:0060089	Molecular transducer activity	7.22E−05	0.092
Function	GO:0038023	Signaling receptor activity	1.32E−04	0.127
Transcripts with lower expression in IA				
Function	GO:0043295	Glutathione binding	1.16E−04	0.148
Function	GO:0046906	Tetrapyrrole binding	1.40E−04	0.134

Gene set enrichment analysis was performed on the 95 significantly differentially expressed genes (q < 0.05) in peripheral blood samples obtained from patients with intracranial aneurysms (IA). Significantly enriched ontologies with a false discovery rate (FDR) adjusted p-value (q-value) < 0.20 were considered (FDR of 20%). Transcripts with higher expression in IA demonstrated regulation of inflammatory and defense responses, signaling, and cell motility. Significantly enriched ontologies in transcripts with lower expression in IA demonstrated regulation of glutathione and tetrapyrrole binding

### Selected transcripts for model training

Prior to model training, we performed feature selection by filtering to identify disease-related transcripts and reduce the data dimensionality to facilitate downstream analysis. Our statistical criteria of false discovery rate (FDR) < 0.05 and an absolute fold-change ≥ 1.5 resulted in the retention of the 26 transcripts that are shown in red in Fig. 1a and listed in Table 3. The PCA in Fig. 1b shows that these 26 transcripts could generally discriminate patients with IAs from the controls. The top three principal components represented 47.8% of the variation: PC1 contained 22.4% variation, PC2 contained 15.3% variation, and PC3 contained 10.1% variation. Overall, 60% of the samples from the IA patients and 80% of those from controls could be grouped together by PCA. Furthermore, our post hoc power analysis estimated that in the 15 vs. 15 training dataset a power of 0.78 was achieved in detecting expression differences of at least 1.5 fold at an α = 0.05. Thus, our feature selection criteria resulting in the identification of the 26 transcripts had a power of > 0.78.

**Table 3:** The 26 transcripts selected for classification model training

Transcript	Gene ID	Accession no.	Log_2_ (F-C)	p-value	q-value
*PVRL2*	5819	NM_002856.2	2.27	5.54E−12	6.94E−09
*CYP1B1*	1545	NM_000104.3	1.53	4.13E−10	3.88E−07
*CD177*	57126	NM_020406.3	1.48	8.04E−06	2.91E−03
*PDE9A*	5152	NM_002606.2	1.45	5.67E−05	9.90E−03
*ARMC12*	221481	NM_145028.4	1.37	1.38E−12	2.07E−09
*OLAH*	55301	NM_018324.2	1.15	1.71E−11	1.83E−08
*TGS1*	96764	NM_024831.7	1.02	1.72E−14	4.31E−11
*CD163*	9332	NM_004244.5	0.98	2.65E−09	1.99E−06
*LOC100506229*	100506229	NR_039975.1	0.96	1.23E−05	3.55E−03
*OCLN*	100506658	NM_002538.3	0.85	4.07E−07	2.37E−04
*SEMA6B*	10501	NM_032108.3	0.80	7.62E−05	1.19E−02
*ADTRP*	84830	NM_001143948.1	0.77	1.61E−05	4.47E−03
*VWA8*	23078	NM_015058.1	0.70	2.56E−06	1.20E−03
*MTRNR2L10*	100463488	NM_001190708.1	0.63	1.21E−05	3.55E−03
*HOXB2*	3212	NM_002145.3	0.62	6.25E−05	1.02E−02
*EPCAM*	4072	NM_002354.2	0.60	1.02E−05	3.50E−03
*IL18R1*	8809	NM_003855.3	0.59	1.17E−05	3.55E−03
*IGSF23*	147710	NM_001205280.1	− 0.80	5.87E−05	9.94E−03
*PTGES*	9536	NM_004878.4	− 0.91	4.78E−05	8.98E−03
*G0S2*	50486	NM_015714.3	− 0.96	6.71E−06	2.66E−03
*FCRL5*	83416	NM_031281.2	− 1.26	4.31E−06	1.80E−03
*C1orf226*	400793	NM_001135240.1	− 1.51	1.27E−14	4.31E−11
*UTS2*	10911	NM_021995.2	− 1.93	8.85E−14	1.66E−10
*HBG2*	3048	NM_000184.2	− 1.97	6.62E−10	5.53E−07
*CYP26B1*	56603	NM_019885.3	− 2.99	4.32E−07	2.37E−04
*C1QL1*	10882	NM_006688.4	− 3.25	5.16E−22	3.88E−18

Significantly differentially expressed transcripts with FDR < 0.05 and absolute fold-change ≥ 1.5. (F-C: fold-change)

### Classification models of IA have high performance in training and testing datasets

Using the expression of these 26 transcripts, we trained the aforementioned four classification models (cosine NN, DLDA, NSC, and SVM). Figure 1c shows the sensitivity, specificity, and accuracy of the models, which were estimated from LOO cross-validation. There was moderate performance by each classification method, with accuracies that ranged from 0.50 to 0.73. Evaluation by ROC curve analysis showed a range in AUCs from 0.54 to 0.73 (Fig. 1d) across all methods. In this dataset, DLDA performed the best, with a sensitivity of 0.67, a specificity of 0.80, an accuracy of 0.73, and an AUC of 0.73.

**Fig. 2:**
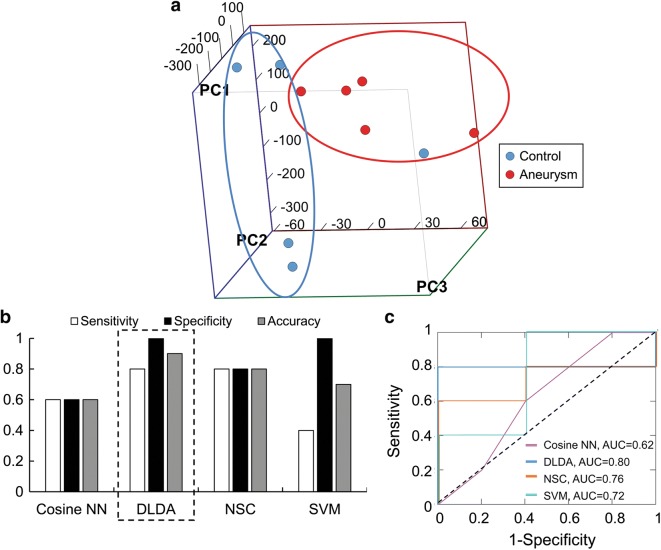
Performance of the four classification models during model testing. **a** PCA using the 26 transcripts showed general separation between patients with IA (100%, circled in red) and controls (80%, circled in blue). **b** Validation of the classification models in an independent testing cohort of patients demonstrated that DLDA had the best performance, with sensitivity, specificity, and accuracy of 0.80, 1.0, and 0.90, respectively. **c** ROC analysis in the testing cohort also showed that DLDA had the best area under the curve (AUC) (0.80)

To independently validate the models, we implemented them in neutrophil transcriptomes from 10 patients in the testing cohort. The PCA in Fig. 2a shows that the 26 transcripts could discriminate patients with IAs from controls in the testing cohort as well. Overall, 100% of the samples from IA patients and 80% of those from controls could be grouped together by PCA. In the testing cohort, the models predicted aneurysm status with a range in accuracy from 0.60 to 0.90 (Fig. 2b). The ROC analysis in Fig. 2c shows that model AUCs ranged from 0.62 to 0.80. In this cohort, the DLDA classification model again performed the best, with a sensitivity of 0.80, specificity of 1.0, an accuracy of 0.90, and an AUC of 0.80.

### Cross-validation to increase model reliability

**Fig. 3:**
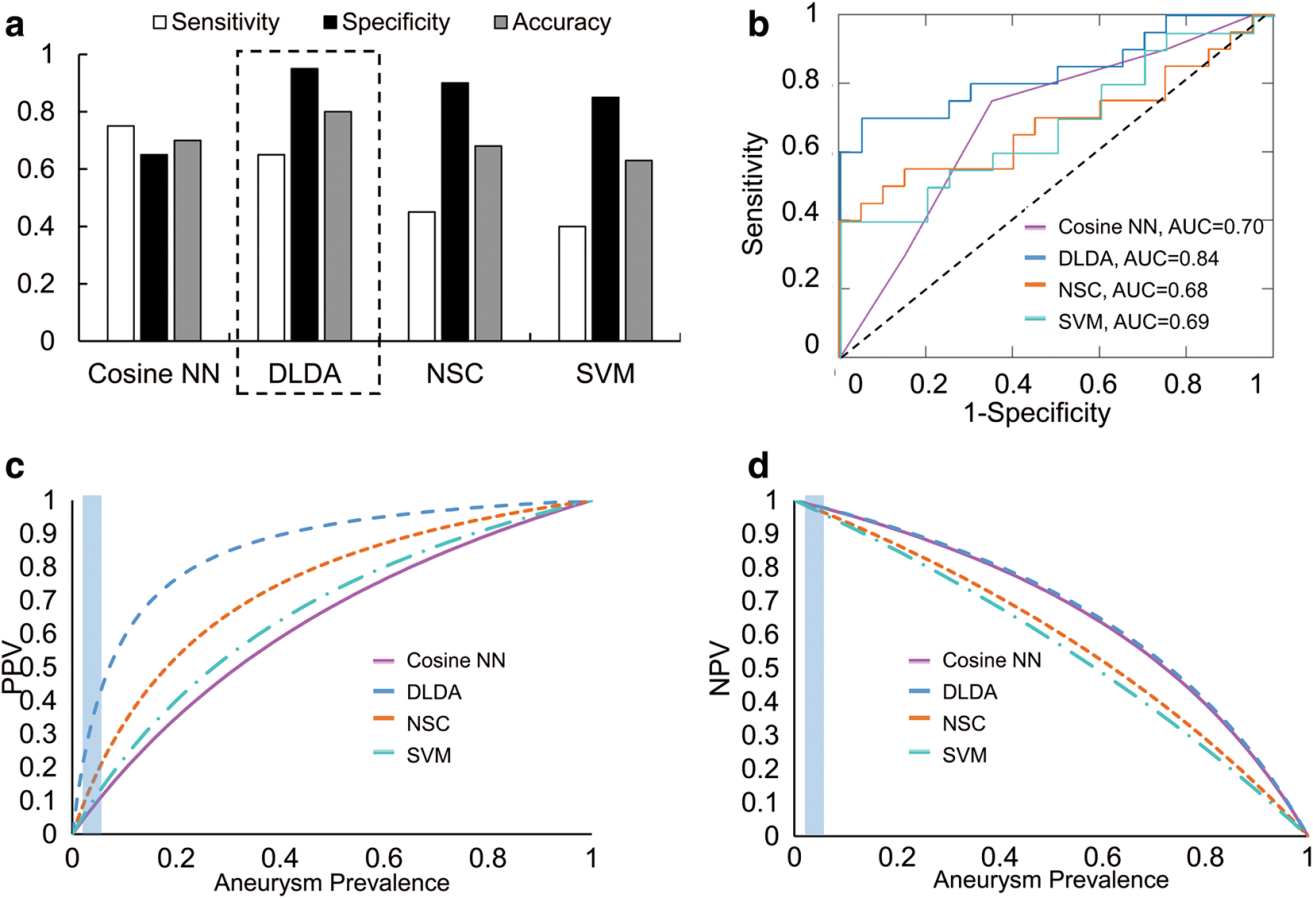
Assessment of model performance by LOO cross-validation of all data, and positive predictive value (PPV)/negative predictive value (NPV). **a** Estimation of model performance showed that the models performed with an accuracy of 0.63–0.80. DLDA had the highest combination of sensitivity, specificity, and accuracy (0.65, 0.95, 0.80, respectively). **b** ROC analysis demonstrated that the models had AUC of 0.68 (NSC) to 0.84 (DLDA). **c** Plot showing the PPV of all models across all possible prevalence. The blue region in the figure represents the range of IA prevalence reported in the current literature. The best performing model (DLDA) had the highest PPV, and cosine NN demonstrated the poorest PPV. **d** The DLDA model also had the best NPV, but only slightly better than that of the cosine NN, NSC, and SVM models

To increase model reliability, we applied LOO cross-validation using all patient transcriptomes and refit them in all 40 datasets (all combinations of 39 training samples and 1 testing sample). This analysis revealed that model accuracy ranged from 0.63 to 0.80 (Fig. 3a) and model AUCs ranged from 0.68 to 0.84 (Fig. 3b). Again, the DLDA model performed the best, with a sensitivity of 0.65, specificity of 0.95, accuracy of 0.80, and an AUC of 0.84.

### Models have high negative predictive value

Given their range of performance, we wanted to know how useful the models would be at detecting IA. However, their value would be inherently influenced by the prevalence of IA in a given target population. To estimate this, we plotted the positive predictive value (PPV) and negative predictive value (NPV) for each model (Fig. 3c, d) using the sensitivity and specificity reported after the LOO cross-validation in all datasets. The rate of aneurysm prevalence found in the literature is between 3.2% [45] to 7% [46], and is highlighted in the blue region on the graphs in Fig. 3c, d. At 5% prevalence, the PPV of the models ranged from 0.10 to 0.41 and NPV ranged from 0.96 to 0.98. The DLDA classifier had the highest PPV (0.41) and NPV (0.98).

### Independent validation of expression differences by RT-qPCR

**Fig. 4:**
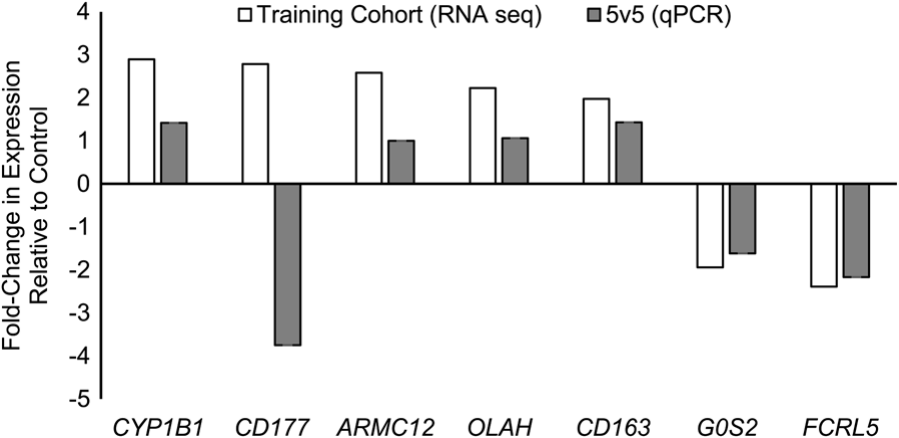
Validation of RNA-Sequencing data for seven transcripts by quantitative polymerase chain reaction (qPCR). Six of seven differentially expressed transcripts in samples from patients with and without IA were also differentially expressed in neutrophils in the qPCR in an independent cohort. This demonstrates consistent expression differences between patients with IA and controls in ~ 86% (6/7) of the tested transcripts

We performed a corroboration study to determine if the differential expression of seven model genes could be detected in a new population of IA and control patients. We used samples from 10 additional patients (5 with IA and 5 controls) from which we collected neutrophil RNA but did not sequence (see Additional file 1: Table S6 for patient information for this cohort and Additional file 1: Table S7 for aneurysm information from the IA patients in this cohort). These samples were used for quantitative qPCR analysis of *CD177*, *CYP1B1*, *ARMC12*, *OLAH*, *CD163*, *G0S2*, and *FCRL5*, which were selected because they were also differentially expressed in our previous study [10]. Figure 4 shows the qPCR results of this corroborative study in comparison with expression differences obtained from RNA sequencing in the training cohort. Six of the seven genes demonstrated average fold-change in the same direction and of similar magnitude to those in the original cohort. This provides evidence that in ~ 86% (6 of 7) of the tested transcripts the expression differences between patients with IA and controls is consistent. This result may serve as the basis for further development of a qPCR-based diagnostic.

### Signals of the classifier genes are stronger for larger IAs

**Fig. 5:**
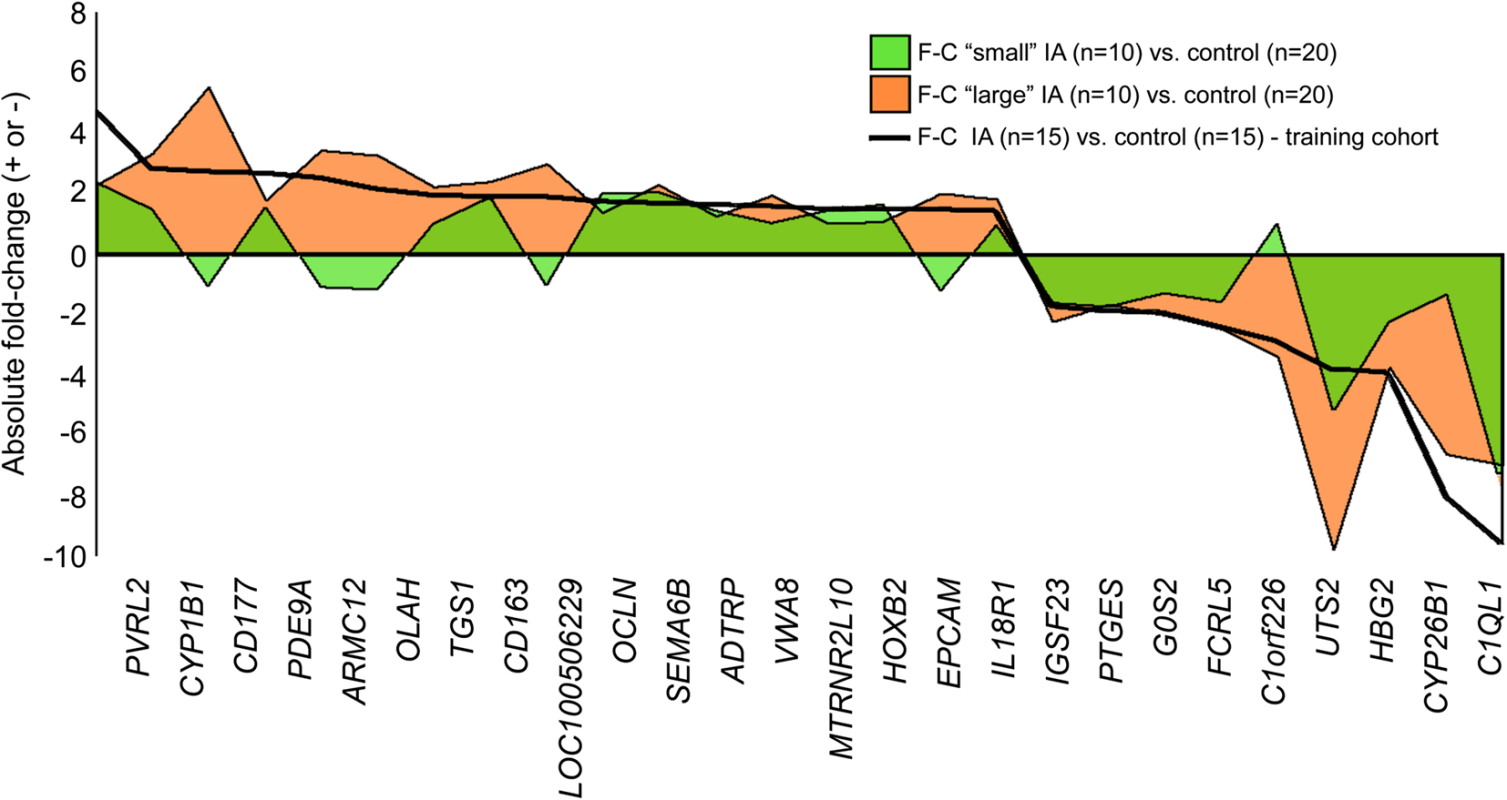
Comparison of fold-change in expression in patients with “small” (< 5 mm) IAs vs. control and patients with “large” (≥ 5 mm) IAs vs. control. The plot shows the fold-change (F-C) in expression of the 26 classifier transcripts identified in the training cohort (n = 30—black line) compared to those for “small” IAs (vs. control—green) and “large” IAs (vs. control—orange). Expression changes were more pronounced in both the positive and negative direction in patients with larger IAs. Fold-changes across all 26 transcripts in the “large” group were on average 24% higher than those for the training cohort, while fold-changes for the “small” group F-C were on average 35% lower

To determine if there was a correlation between aneurysm size and differential expression of the 26 neutrophil classifier transcripts, we separately compared expression levels in patients with “small” and “large” IAs to those in all control patients (n = 20). We used the 5 mm aneurysm size cutoff [51], which dichotomized the IA cohort (n = 20) into a 10-patient “small” group and a 10-patient “large” group. Figure 5 shows the fold-changes between the “small” group and control samples (green) and the “large” group and control samples (orange) for each of the 26 classifier transcripts. These are compared to the fold-changes between aneurysm and control samples in the entire training cohort as a baseline (solid black line). Expression changes were more pronounced in both the positive and negative direction in patients with larger IAs. On average, the fold-change was 24% greater than the baseline for the “large” group, but 35% lower than baseline for the “small” group.

## Discussion

Neutrophils play a role in the progressive inflammation that typifies IAs [57]. We hypothesized that gene expression patterns in circulating neutrophils could indicate the presence of aneurysm. Previously, we found that patients with unruptured IAs and aneurysm-free controls had significant RNA expression differences in circulating neutrophils [10]. In the present study, we tested the feasibility of developing biomarkers using neutrophil RNA expression levels from blood samples to predict IA presence. Using RNA expression profiling in circulating neutrophils, we identified 26 transcripts that were highly associated with the presence of IA. Machine-learning algorithms were then implemented to develop classification models to test whether these 26 transcripts could predict the presence of an aneurysm.

### Classification models of IA using neutrophil RNA expression

To our knowledge, this is the first to demonstrate IA prediction from RNA expression patterns in the blood. The prediction models we trained had a classification accuracy of up to 90% in the test dataset, a level that indicates promise for further investigation of RNA expression biomarkers for IA. Overall, classification by DLDA achieved the best performance in our data. This model consistently had the highest accuracy and AUC over multiple analyses, including cross-validation during model training (accuracy = 0.73, AUC = 0.73), independent model testing (accuracy = 0.90, AUC = 0.80) and cross-validation across the entire dataset (accuracy = 0.80, AUC = 0.84). See Additional file 1: Table S8 for a summary of the performance of all four models.

We suspect that DLDA outperformed the other methods because it best accounted for potential intersample variability of the 26 transcripts. Modeling techniques that broadly survey patterns of gene expression may afford better IA classification [58, 59]. The DLDA method does this by (1) ranking transcripts by importance, giving more weight to the most consistently informative transcripts (unlike non-parametric approaches such as NN); and (2) using information from all transcripts to project test samples to the direction which best separates the classes. In this way, a linear combination of transcripts, which may be individually inconsistent, can generate a stable IA prediction and accommodate for potential intersample variability. Additionally, ignoring correlations between genes as DLDA does, provided a simple model and produced lower misclassification rates than more sophisticated classifiers, such as SVM.

In the current study, classifiers were developed based on 30 transcriptomes that were randomly selected from all available data (n = 40). Randomization was used so we could test the viability of IA prediction in patients who have potentially confounding covariates (comorbidities and demographics). Table 1 shows differences in smoking history between the IA and control groups in the training cohort, which may reflect an established association between the presence of an IA and smoking [60]. It is worth noting that this study was designed differently from our previous investigation [10]. There we identified an 82-transcript expression signature of IA by transcriptome profiling of a cohort-controlled group of patients, whereas in this study the randomly selected training cohort was not cohort-controlled. Yet, even with this difference, 10 of the 26 classifier transcripts (38%) were also a part of the previously discovered 82-transcript signature. These genes include *CYP1B1*, *CD177*, *ARMC12*, *OLAH*, *CD163*, *ADTRP*, *VWA8*, *G0S2*, *FCRL5*, and *C1orf226*. Notably, in qPCR validation on seven of these genes, six of them (*CYP1B1, ARMC12, OLAH, CD163, G0S2,* and *FCRL5*) showed consistent expression differences. These six transcripts may warrant further investigation as they may be most important for IA detection.

### Biological role of classifier transcripts

The natural history of IA has been characterized by mounting inflammatory responses accompanied by progressive degradation of the aneurysmal wall [61, 62]. This begins during aneurysm initiation, in which a combination of risk factors and hemodynamic stresses elicit pro-inflammatory changes in smooth muscle cells that lead to overproduction of matrix metalloproteinases (MMPs) that degrade the arterial extracellular matrix [61, 62]. Once the aneurysmal sac forms, recirculating blood in the IA is conducive to inflammatory cell infiltration into the wall, which is also assisted by an increase of chemokines and cytokines in both the aneurysm wall and in the plasma within the aneurysm [63, 64]. Recruited inflammatory infiltrates also produce MMPs that continue to degrade the aneurysm wall and advance its growth and rupture [57, 62], which can ultimately occur when the wall is weakened to the point that it can no longer contain the force of the blood pressure [61]. This is demonstrated by histological analyses and gene expression studies of human aneurysmal tissues, which have found increased matrix degradation proteins, inflammatory processes, and inflammatory cytokines and chemoattractant proteins in the walls of aneurysms [65, 66].

Despite being the most abundant circulating immune cell, the role that neutrophils play in IA pathophysiology is relatively unknown. However, since neutrophils are recruited to sites of injury to coordinate the inflammatory response and attract inflammatory cells (monocytes) during other vascular pathologies [67], we initially suspected they may play a similar role in IA. Studies suggest that neutrophils reside in intraluminal thrombi that have formed on the wall of the aneurysmal sac [68, 69]. There, besides secreting MMP-9, activated neutrophils can release myeloperoxidase (MPO) and neutrophil gelatinase associated lipocalin (NGAL) that can indirectly promote extracellular matrix degradation and cytotoxicity. Elevated levels of MPO, a peroxidase enzyme secreted during degranulation, provoke pro-inflammatory cell signaling and oxidative stress via increased production of reactive oxygen species [70]. Increased NGAL protects MMP-9 from degradation, thereby increasing its activity and promoting wall degeneration [64]. Interestingly, levels of MPO and NGAL have been shown to be elevated in the peripheral blood of patients with IAs [64, 71], which can act in an autocrine manner to promote activation of circulating neutrophils [72, 73]. In this study, however, we did not observe significantly higher expression of the MPO or NGAL genes in circulating neutrophils, which suggests that the source of these circulating proteins could be the wall itself or other blood cells.

Our data shows that circulating neutrophils from patients with IA are peripherally activated. From gene ontology analysis on all 95 differentially expressed genes (q < 0.05), we found that they have dysregulated inflammatory and defense responses, and increased signaling and response to stimuli. Increased IL-1 signal transduction through receptors *IL1R1* and *IL1R2* has been shown to play a major role in neutrophil activation [74–76]. Increased neutrophil activation was also evidenced through greater expression levels of several CD antigens, namely *CD36*, *CD99L2*, *CD163*, and *CD177.* Specifically, *CD177* is a marker of neutrophil activation that plays a role in migration [77], and *CD99L2* is involved in neutrophil recruitment to inflamed tissues [78]. These findings are consistent with the results of our previous cohort-controlled study [10], which also showed increased peripheral leukocyte activation in neutrophils from IA patients.

In the subset of the 26 classifier transcripts, neutrophil activation was reflected through the roles of five genes (*CD177*, *IL18R1*, *PVRL2*, *PDE9A*, and *PTGES*). Like *CD177*, *IL18R1* has been shown to be involved in neutrophil activation as well as migration via IL-18 signaling [79]. *Nectin*-*2* (*PVRL2*), a membrane glycoprotein, is involved in cell adhesion [80], and has been shown to have increased expression in the aneurysm wall tissue [81]. Similarly, *PDE9A* (a cGMP-specific phosphodiesterase) is also involved in cell adhesion [80, 82] and, as demonstrated by Li et al. [83], is regulated by two of the most active transcription factors in the IA tissue. Furthermore, lower *PTGES* expression may be partially responsible for increasing the lifespan of neutrophils, because it is involved in p53-induced apoptosis [84]. We suspect that capturing neutrophil activation responses involved in IA is the reason why the 26-transcript biomarker was able to detect IA.

Besides these five genes, nine other transcripts (*CD163, TGS1, CYP26B1, ADTRP, OCLN, OLAH*, *C1QL1, FCRL5,* and *IGSF23*) in the 26-transcript classifier reflect complex inflammatory processes, albeit not specifically attributed to neutrophil activation. For example, *CD163*, which is abundant in the walls of IAs (but typically associated with macrophages [85, 86]), has been shown to be increased in neutrophils during sepsis [87] and thus could be contributing to vascular inflammation in IA. Expression differences of other transcripts, like *TGS1* and *CYP26B1* (that are differentially expressed in tuberculosis [88] and juvenile idiopathic arthritis [12], respectively) could be related to neutrophil responses to intravascular perturbations during IA. Other transcripts—such as *ADTRP* (expressed by macrophages in coronary artery plaques) [89], *OCLN* (increased in activated T-lymphocytes and in whole blood during sepsis) [90, 91], *OLAH* (increased in peripheral blood mononuclear cells during non-small cell lung cancer) [92], *C1QL1* (a complement component decreed in glioblastoma multiform) [93], *FCRL5* (an immunoglobulin receptor that regulates B cell response to antigen) [94], and *IGSF23* (decreased during the inflammatory response associated with mycoestrogen exposure) [95]—are involved in inflammation but have not been reported to be differentially expressed in neutrophils. The roles of the remaining model transcripts (e.g., *C1orf226*, *LOC100506229*, *MTRNR2L10*) in neutrophils are unknown. Further study into the roles of these transcripts in IA may be warranted, as they could represent novel predictive targets in neutrophil RNA expression.

Taken together, our results suggest that circulating neutrophils are peripherally activated in patients with IA, which leads to a change in their RNA expression profile. We postulate this activation could be caused by contact with inflamed aneurysm tissue that often contains denuded regions and plaque or thrombus [96–98]. Alternatively, the activation may be caused by chemokines and cytokines released from the aneurysm. Chalouhi et al. [63] demonstrated that blood inside IAs contain increased concentrations of the chemokines MCP-1, RANTES, MIG, IP-10, and exotoxin, and chemoattractant cytokines, including IL-8 and IL-17. Either of the above two scenarios may explain why expression differences of the 26 classifier transcripts were more pronounced in patients with larger IAs, since larger IAs provide greater luminal surface area for either contact or release of inflammatory mediators. It would be interesting to conduct a longitudinal study of patients with growing aneurysms to ascertain the relationship between aneurysm development and the effect on gene expression in circulating neutrophils.

### Limitations

Due to our small sample size, the results in this study are rather preliminary. However, we have confidence in the discovered classifier transcripts for the following reasons. (a) The classifier identified patients with IA in an independent testing cohort with 90% accuracy. (b) qPCR confirmed expression differences in an independent validation cohort. (c) Our post hoc power analysis demonstrated > 0.78 power. In the future, we could further increase reliability in the model transcripts by decreasing the number of features in the data and increasing sample size.

Secondly, our classifier was derived from a population of patients who had different rates of demographic factors and comorbidities between aneurysm and control patients. It is unclear whether the presence of these confounding factors contributes to the differential neutrophil expression we detected. We are currently conducting research on larger cohorts by including multiple control groups with different vascular pathologies (including extracranial aneurysms such as abdominal aortic aneurysm) and immunological conditions (both of which were excluded in the current study) to narrow down transcripts specific to IA. Finally, although we collected basic demographics and information about comorbidities, including hypertension and diabetes, there could be others factors, such as the presence of immune-metabolism mediators in the blood that could affect gene expression in circulating neutrophils. Efforts should be made to collect higher fidelity patient health metadata.

## Conclusions

We have shown for the first time the feasibility of using blood-based biomarkers to detect unruptured IA. A model consisting of 26 transcripts predicted IA presence with an accuracy of 0.80 and an AUC of 0.84. This biomarker reflects gene expression changes due in part to activation of circulating neutrophils associated with IA. Pending confirmation in larger cohorts, these results suggest an exciting potential to develop blood-based IA tests to screen for IA in high-risk populations.

## Additional file

**Additional file 1.** Additional Tables S1–S8.

## Abbreviations

AUC: area under the receiver operating characteristic curve
cosine NN: cosine nearest neighbors
DLDA: diagonal linear discriminant analysis
DSA: digital subtraction angiography
FDR: false discovery rate
FN: false negative
FP: false positive
FPKM: fragments per kilobase million
GO: gene ontology
GORILLA: Gene Ontology enRIchment anaLysis and visuaLizAtion tool
IA: intracranial aneurysm
LDA: linear discriminant analysis
LOO: leave-one-out
NN: nearest neighbors
NPV: negative predictive value
NSC: nearest shrunken centroids
PC: principal component
PCA: principal component analysis
PPV: positive predictive value
qPCR: quantitative polymerase chain reaction
ROC: receiver operator characteristic
SVM: support vector machines
TN: true negative
TP: true positive
TPM: transcripts per million
