# Supplementary material for: Biomarkers from circulating neutrophil transcriptomes have potential to detect unruptured intracranial aneurysms
Source: J Transl Med. 2018 Dec 28;16:373. doi: 10.1186/s12967-018-1749-3 (PMC6310942; doi:10.1186/s12967-018-1749-3)
Supplement: Supplementary file 1 — Additional file 1. Additional Tables S1–S8. [file 12967_2018_1749_MOESM1_ESM.docx]

**Additional Material for:**

**Biomarkers from circulating neutrophil transcriptomes have potential to detect unruptured intracranial aneurysms**

**Authors**: Vincent M. Tutino PhD^1,2^, Kerry E. Poppenberg BS^1,2^, Lu Li MS^3^, Hussain Shallwani MD^4^, Kaiyu Jiang PhD^5^, James N. Jarvis MD^5,6^, Yijun Sun PhD^5,7^, Kenneth V. Snyder MD PhD^1,4,8,9^, Elad I. Levy MD MBA^1,4,8^, Adnan H. Siddiqui MD PhD^1,4,^, John Kolega PhD^1,10^, *Hui Meng PhD^1,2,4,11^

**Affiliations**: ^1^Canon Stroke and Vascular Research Center; ^2^Department of Biomedical Engineering, ^3^Department of Computer Science and Engineering,  ^4^Department of Neurosurgery, Jacobs School of Medicine and Biomedical Sciences,  ^5^Genetics, Genomics, and Bioinformatics Program, ^6^Department of Pediatrics, Jacobs School of Medicine and Biomedical Sciences, ^7^Department of Microbiology and Immunology, ^8^Department of Radiology, Jacobs School of Medicine and Biomedical Sciences, ^9^Department of Neurology, Jacobs School of Medicine and Biomedical Sciences,  ^10^Department of Pathology and Anatomical Sciences, Jacobs School of Medicine and Biomedical Sciences, ^11^Department of Mechanical & Aerospace Engineering, University at Buffalo; Buffalo, New York, USA

***Correspondence:**

Hui Meng, PhD

Canon Stroke and Vascular Research Center

Clinical and Translational Research Center

875 Ellicott Street

Buffalo, NY 14214 USA

E-mail:[huimeng@buffalo.edu](mailto:huimeng@buffalo.edu); Phone:(716)829-5400; Fax: (716)854-1850

**Table S1. Batch Assignment.***

|  | **ID** | **Class** | **Batch** |  | |
| --- | --- | --- | --- | --- | --- |
| ***Training Cohort*** | | | | | |
|  | C1 | Control | 1 | |  |
|  | C2 | Control | 1 | |  |
|  | C3 | Control | 1 | |  |
|  | C4 | Control | 1 | |  |
|  | C5 | Control | 1 | |  |
|  | C6 | Control | 1 | |  |
|  | C7 | Control | 1 | |  |
|  | C8 | Control | 2 | |  |
|  | C9 | Control | 2 | |  |
|  | C10 | Control | 2 | |  |
|  | C11 | Control | 2 | |  |
|  | C12 | Control | 2 | |  |
|  | C13 | Control | 2 | |  |
|  | C14 | Control | 2 | |  |
|  | C15 | Control | 2 | |  |
|  | A1 | Aneurysm | 1 | |  |
|  | A2 | Aneurysm | 1 | |  |
|  | A3 | Aneurysm | 1 | |  |
|  | A4 | Aneurysm | 1 | |  |
|  | A5 | Aneurysm | 1 | |  |
|  | A6 | Aneurysm | 1 | |  |
|  | A7 | Aneurysm | 1 | |  |
|  | A8 | Aneurysm | 1 | |  |
|  | A9 | Aneurysm | 2 | |  |
|  | A10 | Aneurysm | 2 | |  |
|  | A11 | Aneurysm | 2 | |  |
|  | A12 | Aneurysm | 2 | |  |
|  | A13 | Aneurysm | 2 | |  |
|  | A14 | Aneurysm | 2 | |  |
|  | A15 | Aneurysm | 1 | |  |
| ***Testing Cohort*** | | | | |  |
|  | C16 | Control | 1 | |  |
|  | C17 | Control | 1 | |  |
|  | C18 | Control | 1 | |  |
|  | C19 | Control | 2 | |  |
|  | C20 | Control | 1 | |  |
|  | A16 | Aneurysm | 1 | |  |
|  | A17 | Aneurysm | 1 | |  |
| A18 | | Aneurysm | 2 | |  |
| A19 | | Aneurysm | 2 | |  |
| A20 | | Aneurysm | 2 | |  |

*Over the study period, samples were collected and processed in 2 main batches. The earlier batch is designated “1,” and the later batch is designated “2.” When creating the training and testing cohorts, data from each batch were randomly partitioned into each group.

**Table S2. Primers used for qPCR and their efficiencies.***

| **Transcript** | **Primer Sequence** | **Annealing**  **Temp. (^o^C)** | **Eff.** | **PCR Prod.**  **Length (bp)** |
| --- | --- | --- | --- | --- |
| *CD177* | 5'-ACACACGGAAACTTGGCTCA-3' | 60.0 | 1.04 | 124 |
|  | 5'-CCAGGGTTGATGTGAGTCCTAC-3' |  |  |  |
| *CYP1B1* | 5'-CAGTGAATTTGGGCTGCTGT-3' | 60.0 | 0.95 | 148 |
|  | 5'-TGACGACTGGGCCTACATAC-3' |  |  |  |
| *ARMC12* | 5'-CCTCAACAACCTTCCACTGCC-3' | 60.0 | 1.04 | 130 |
|  | 5'-CAGGTAGCTCAGCAGTCGTA-3' |  |  |  |
| *OLAH* | 5'-GCAGCCAGTCATCCAGGATA-3' | 60.0 | 0.92 | 175 |
|  | 5'-ATCTTTGGGAATGCGATGCC-3' |  |  |  |
| *CD163* | 5'- TGTCGTGGGAATGAGTCAGC-3' | 60.0 | 0.99 | 109 |
|  | 5'- TGGATCCATCTGAGCAGGTC-3' |  |  |  |
| G0S2 | 5'-CACTAAGGTCATTCCCGCCT-3' | 60.0 | 0.91 | 125 |
|  | 5'-AGCACGTACAGCTTCACCAT-3' |  |  |  |
| FCRL5 | 5'-TCAGTGTCTACCTGCCCAAG-3' | 60.0 | 1.07 | 89 |
|  | 5'-GCCTTGACTTGCTGGGTTAC-3' |  |  |  |
| GAPDH | 5'-CGCTCTCTGCTCCTCCTGTT-3' | 60.0 | 1.09 | 81 |
|  | 5'-CCATGGTGTCTGAGCGATGT-3' |  |  |  |
| 18s rRNA | 5'-GGCCCTCTAATTGGAAGAGTC-3' | 60.0 | 1.09 | 145 |
|  | 5'-CCAAGATCCAACTACGAGCTT-3' |  |  |  |
| GPI | 5'-AGGCTGCTGCCACATAAGGT-3' | 60.0 | 0.95 | 240 |
|  | 5'-AGCGTCGTGAGAGGTCACTTG-3' |  |  |  |

*Primers were selected using Primer3 and NCBI’s Primer Blast. All efficiencies were within the range of 0.90–1.10. (bp=base pair, Eff.=efficiency, Prod.=product, qPCR=quantitative polymerase chain reaction, Temp.=temperature)

**Table S3. RNA Quality.***

|  | **ID** | **Class** | **260/280** | **RIN** | |  |
| --- | --- | --- | --- | --- | --- | --- |
| ***Training Cohort*** | | | | | |  |
|  | C1 | Control | 2.02 | | 7.4 |  |
|  | C2 | Control | 1.92 | | 6.5 |  |
|  | C3 | Control | 2.08 | | 7.1 |  |
|  | C4 | Control | 2.05 | | 7.9 |  |
|  | C5 | Control | 1.96 | | 6.7 |  |
|  | C6 | Control | 2.08 | | 6.2 |  |
|  | C7 | Control | 2.04 | | 7.3 |  |
|  | C8 | Control | 2.04 | | 6.6 |  |
|  | C9 | Control | 2.04 | | 6.0 |  |
|  | C10 | Control | 2.05 | | 6.0 |  |
|  | C11 | Control | 2.10 | | 5.5 |  |
|  | C12 | Control | 2.01 | | 7.9 |  |
|  | C13 | Control | 2.03 | | 6.0 |  |
|  | C14 | Control | 1.90 | | 5.2 |  |
|  | C15 | Control | 2.02 | | 8.2 |  |
|  | A1 | Aneurysm | 2.04 | | 7.8 |  |
|  | A2 | Aneurysm | 2.07 | | 7.5 |  |
|  | A3 | Aneurysm | 2.02 | | 6.1 |  |
|  | A4 | Aneurysm | 2.03 | | 7.3 |  |
|  | A5 | Aneurysm | 1.99 | | 6.5 |  |
|  | A6 | Aneurysm | 2.02 | | 7.5 |  |
|  | A7 | Aneurysm | 2.05 | | 7.7 |  |
|  | A8 | Aneurysm | 1.95 | | 7.2 |  |
|  | A9 | Aneurysm | 2.07 | | 6.7 |  |
|  | A10 | Aneurysm | 2.00 | | 6.9 |  |
|  | A11 | Aneurysm | 1.97 | | 6.4 |  |
|  | A12 | Aneurysm | 2.12 | | 7.3 |  |
|  | A13 | Aneurysm | 1.96 | | 7.4 |  |
|  | A14 | Aneurysm | 2.07 | | 7.8 |  |
|  | A15 | Aneurysm | 1.97 | | 6.9 |  |
| ***Testing Cohort*** | | | | | |  |
|  | C16 | Control | 2.07 | | 7.0 |  |
|  | C17 | Control | 2.05 | | 6.6 |  |
|  | C18 | Control | 1.99 | | 7.1 |  |
|  | C19 | Control | 1.92 | | 6.6 |  |
|  | C20 | Control | 1.97 | | 6.4 |  |
|  | A16 | Aneurysm | 2.06 | | 8.1 |  |
|  | A17 | Aneurysm | 2.07 | | 6.0 |  |
|  | A18 | Aneurysm | 2.08 | | 7.1 |  |
|  | A19 | Aneurysm | 2.03 | | 7.2 |  |
|  | A20 | Aneurysm | 2.06 | | 6.0 |  |

* The quality of the RNA samples was assessed by the 260/280 ratio and the RIN. (RIN=RNA integrity number)

**Table S4**. **Characteristics of 27 aneurysms in all patients with intracranial aneurysms (5 patients had multiple intracranial aneurysms)***

| **ID** | **IA**  **Size** | **IA**  **Location** | **Presence of**  **Additional IAs** | **Family**  **History of IA** | **Indications for DSA** |  |
| --- | --- | --- | --- | --- | --- | --- |
| ***Training Cohort*** | | | | | | |
| A1 | 10 mm | VB Junction | No | No | MRI for hand numbness indicated possible IA | |
| A2 | 8 mm | ICA Paraophthalmic | No | No | Follow-up imaging of known IA | |
| A3 | 4 mm | Ophthalmic | +2 (1.5 mm ICA, 3 mm clinoid segment) | No | MRI for headache indicated possible IA | |
| A4 | 10.8 mm | MCA | +2 (2.3 mm MCA, small AComA) | No | Incidental finding on MRI indicated possible IA | |
| A5 | 9 mm | PComA | No | No | Follow-up of known IA | |
| A6 | 5 mm | BT | No | No | MRA and CT for tremor revealed possible IA | |
| A7 | 13 mm | ACA | No | Yes | MRI for decreased vision in left eye indicated possible IA | |
| A8 | 5 mm | ICA | +1 (3.5 mm paraophthalmic) | Yes | MRI for tremors indicated possible IA | |
| A9 | 6 mm | MCA | No | No | MRA following vehicle accident indicated possible IA | |
| A10 | 3.7 mm | AComA | No | No | Incidental finding on CT for headache indicated possible IA | |
| A11 | 1.4 mm | MCA | No | No | Incidental finding on MRI indicated possible IA | |
| A12 | 2 mm | PCA | +1 (2mm ICA) | No | MRI for migraine indicated possible IA | |
| A13 | 3.9 mm | BT | No | No | Incidental finding on MRA for headache indicated possible IA | |
| A14 | 1 mm | ICA | No | Yes | Follow-up imaging of known IA | |
| A15 | 3 mm | BT | No | No | MRI for headache indicated possible IA | |
| ***Testing Cohort*** | | | | | | |
| A16 | 4.5 mm | MCA | No | No | Incidental finding on CT indicated possible IA | |
| A17 | 19 mm | ICA | No | No | MRI for double vision indicated possible IA | |
| A18 | 5.1 mm | ICA | No | No | MRI for headache indicated possible IA | |
| A19 | 7 mm | MCA | +1 (3.5 mm ACA) | No | Follow-up imaging of known IA | |
| A20 | 2 mm | ACA | No | No | Follow-up imaging of known IA | |

*Intracranial aneurysm (IA) size ranged from 1 mm to 19 mm in greatest diameter. Sixteen IAs (60%) were classified as small (diameter <5 mm), and 11 (40%) were classified as large (diameter ≥5 mm). The aneurysms were situated at various locations in the Circle of Willis, with most around the internal carotid artery (ICA) and its branches. Three patients with IAs had a family history of the disease. In general, digital subtraction angiography was performed for confirmation of IA presence after an incidental finding of IA on noninvasive imaging or for follow-up imaging of a previously detected IA. (ACA=anterior cerebral artery, AComA=anterior communicating artery, BT=basilar terminus, CT=computed tomography, DSA=digital subtraction angiography, IA=intracranial aneurysm, ICA=internal carotid artery, MCA=middle cerebral artery, MRA=magnetic resonance angiography, MRI=magnetic resonance imaging, PComA=posterior communicating artery, VB=vertebrobasilar)

**Table S5. RNA Sequencing Quality Control Analysis.***

|  |  |  | |  | | | | | |  | |  |  |
| --- | --- | --- | --- | --- | --- | --- | --- | --- | --- | --- | --- | --- | --- |
|  | **ID** | **Class** | | **M. Seqs.** | | **Poor Qual. Seqs.** | | **Seqs. Length** | **% GC** | **% Aligned** | **M. Aligned** | | **Detected Transcripts** |
| ***Training Cohort*** | | | | | |  | | |  | |  | |  |
|  | C1 | | Control | | 59.3 | 0 | 51 | | 49 | 96.50% | 54.4 | | 11434 |
|  | C2 | | Control | | 68.9 | 0 | 51 | | 49 | 96.60% | 63.3 | | 11928 |
|  | C3 | | Control | | 80.3 | 0 | 51 | | 50 | 96.30% | 73.5 | | 11829 |
|  | C4 | | Control | | 97.4 | 0 | 51 | | 50 | 96.00% | 88.9 | | 11064 |
|  | C5 | | Control | | 67.8 | 0 | 51 | | 49 | 96.40% | 62.2 | | 12135 |
|  | C6 | | Control | | 36.2 | 0 | 51 | | 49 | 96.10% | 33.2 | | 12057 |
|  | C7 | | Control | | 79.3 | 0 | 51 | | 49 | 96.80% | 72.7 | | 10117 |
|  | C8 | | Control | | 66.0 | 0 | 51 | | 50 | 95.50% | 60.0 | | 12048 |
|  | C9 | | Control | | 74.4 | 0 | 51 | | 50 | 95.10% | 66.8 | | 12154 |
|  | C10 | | Control | | 64.3 | 0 | 51 | | 51 | 94.90% | 57.9 | | 11866 |
|  | C11 | | Control | | 36.9 | 0 | 51 | | 51 | 96.30% | 33.7 | | 11072 |
|  | C12 | | Control | | 51.5 | 0 | 51 | | 50 | 95.60% | 46.8 | | 11823 |
|  | C13 | | Control | | 58.5 | 0 | 51 | | 51 | 95.50% | 53.0 | | 11779 |
|  | C14 | | Control | | 64.6 | 0 | 51 | | 50 | 94.10% | 57.7 | | 11304 |
|  | C15 | | Control | | 47.3 | 0 | 51 | | 50 | 95.30% | 42.7 | | 12191 |
|  | A1 | | Aneurysm | | 48.1 | 0 | 51 | | 49 | 96.90% | 44.5 | | 11656 |
|  | A2 | | Aneurysm | | 35.7 | 0 | 51 | | 48 | 96.80% | 32.9 | | 11101 |
|  | A3 | | Aneurysm | | 55.9 | 0 | 51 | | 49 | 96.90% | 51.4 | | 11725 |
|  | A4 | | Aneurysm | | 61.3 | 0 | 51 | | 49 | 97.20% | 56.9 | | 11087 |
|  | A5 | | Aneurysm | | 23.4 | 0 | 51 | | 49 | 95.90% | 21.4 | | 11951 |
|  | A6 | | Aneurysm | | 35.8 | 0 | 51 | | 49 | 96.40% | 32.3 | | 12108 |
|  | A7 | | Aneurysm | | 26.9 | 0 | 51 | | 50 | 97.10% | 24.9 | | 10151 |
|  | A8 | | Aneurysm | | 59.5 | 0 | 51 | | 49 | 96.00% | 54.1 | | 12111 |
|  | A9 | | Aneurysm | | 86.9 | 0 | 51 | | 51 | 84.60% | 70.0 | | 10809 |
|  | A10 | | Aneurysm | | 64.9 | 0 | 51 | | 50 | 96.60% | 59.5 | | 10869 |
|  | A11 | | Aneurysm | | 75.3 | 0 | 51 | | 51 | 95.90% | 68.6 | | 11938 |
|  | A12 | | Aneurysm | | 47.2 | 0 | 51 | | 51 | 89.50% | 39.9 | | 11512 |
|  | A13 | | Aneurysm | | 42.1 | 0 | 51 | | 50 | 95.20% | 38.2 | | 11185 |
|  | A14 | | Aneurysm | | 32.3 | 0 | 51 | | 50 | 88.50% | 27.2 | | 11903 |
|  | A15 | | Aneurysm | | 14.6 | 0 | 51 | | 49 | 95.00% | 13.0 | | 12127 |
| ***Testing Cohort*** | | | | | |  | | |  | |  | |  |
|  | C16 | Control | | | 27 | 0 | 51 | | 48 | 96.50% | 24.8 | | 11433 |
|  | C17 | Control | | | 35.9 | 0 | 51 | | 49 | 94.90% | 32.3 | | 12158 |
|  | C18 | Control | | | 53.3 | 0 | 51 | | 50 | 95.80% | 48.5 | | 11626 |
|  | C19 | Control | | | 44.2 | 0 | 51 | | 49 | 96.00% | 40.6 | | 12115 |
|  | C20 | Control | | | 89.1 | 0 | 51 | | 50 | 95.70% | 81.1 | | 11580 |
|  | A16 | Aneurysm | | | 60.4 | 0 | 51 | | 49 | 96.90% | 55.9 | | 12063 |
|  | A17 | Aneurysm | | | 29.1 | 0 | 51 | | 50 | 96.60% | 26.8 | | 11442 |
|  | A18 | Aneurysm | | | 55.6 | 0 | 51 | | 51 | 93.90% | 49.3 | | 10817 |
|  | A19 | Aneurysm | | | 39.8 | 0 | 51 | | 50 | 97.40% | 36.8 | | 11347 |
|  | A20 | Aneurysm | | | 42.2 | 0 | 51 | | 51 | 95.50% | 38.3 | | 12018 |

* Quality of the RNA sequencing experiments. Overall, prior to alignment, all samples had an average of 53.84 M sequences. The sequencing experiments had an average of 48.4 M mapped reads with a 95.37% read mapping rate and detected an average of 11,591 transcripts (transcripts with TPM>1 after batch effect correction). (M.=million, Qual.=quality, Seqs.=sequences, TPM=transcripts per million)

**Table S6. Clinical characteristics of the additional cohort of 5 patients with intracranial aneurysms and 5 control subjects without intracranial aneurysms (confirmed on imaging) used for qPCR validation.***

|  | **Patients with IA**  **(n=5)** | **Control Patients**  **without IA (n=5)** |
| --- | --- | --- |
| **Age, years (Mean ± SE)** | 54.6±3.60 | 50.6±6.23 |
| **Age, years [Median (Q1/Q3)]** | 58 (46/60) | 50 (39/62) |
| **Sex** |  |  |
| *Female* | 40% | 80% |
| **Smoking Status** |  |  |
| *Yes* | 60% | 0% |
| **Comorbidities** |  |  |
| *Hypertension* | 0% | 20% |
| *High Cholesterol* | 60% | 40% |
| *Heart Disease* | 20% | 0% |
| *Stroke History* | 20% | 40% |
| *Diabetes* | 0% | 20% |
| *Osteoarthritis* | 20% | 20% |

* Clinical information, with the exception of age, was quantified as binary data points. Clinical factors were retrieved from the patients’ medical records via the latest “Patient Medical History” form administered prior to imaging. (IA=intracranial aneurysm, Q=quartile, qPCR=quantitative polymerase chain reaction, SE=standard error)

**Table S7**. **Characteristics of intracranial aneurysms in the group of 5 patients with IAs used for qPCR validation.***

| **IA Patient no.** | **IA**  **Size**  **(mm)** | **IA**  **Location** | **Presence of**  **Additional IAs** | **Family**  **History of IA** | **Indications for DSA** |
| --- | --- | --- | --- | --- | --- |
| A21 | 7 | MCA | No | No | Noninvasive imaging for extremity weakness indicated possible IA |
| A22 | 10 | MCA | No | No | Noninvasive imaging seizure indicated possible IA |
| A23 | 6.1 | ICA | No | Yes | Screening due to family history of IA |
| A24 | 2.8 | ICA | No | No | MRI for migraine indicated possible IA |
| A25 | 3.2 | MCA | No | No | CTA imaging indicated possible IA |

*Aneurysm size ranged from 2.8 mm to 10 mm. Two of the 5 IAs (40%) were classified as small (<5mm) and three (60%) were classified as large (≥5 mm). The aneurysms were situated in the Circle of Willis, in the anterior vasculature (MCA) and at the ICA. (CTA=computed tomography angiography, IA=intracranial aneurysm, ICA=internal carotid artery, MCA=middle cerebral artery, MRI=magnetic resonance imaging, qPCR=quantitative polymerase chain reaction)

**Table S8. Summary of classification model performance across different analyses.***

| ***Model training (LOO cross-validation)*** | | | | | |  |  |
| --- | --- | --- | --- | --- | --- | --- | --- |
|  | **Sen.** | | **Spec.** | **Acc.** | **AUC** | **PPV*** | **NPV*** |
| *Cosine NN* | 0.87 | | 0.60 | 0.73 | 0.72 | 0.10 | 0.99 |
| *DLDA* | 0.67 | | 0.80 | 0.73 | 0.73 | 0.15 | 0.98 |
| *NSC* | 0.53 | | 0.93 | 0.73 | 0.73 | 0.28 | 0.97 |
| *SVM* | 0.47 | | 0.53 | 0.50 | 0.54 | 0.05 | 0.95 |
|  |  | |  |  |  |  |  |
| ***Model testing*** | | | |  |  |  |  |
|  | **Sen.** | | **Spec.** | **Acc.** | **AUC** | **PPV*** | **NPV*** |
| *Cosine NN* | 0.60 | | 0.60 | 0.60 | 0.62 | 0.07 | 0.97 |
| *DLDA* | 0.80 | | 1.00 | 0.90 | 0.80 | 1.00 | 0.99 |
| *NSC* | 0.80 | | 0.80 | 0.80 | 0.76 | 0.17 | 0.99 |
| *SVM* | 0.40 | | 1.00 | 0.70 | 0.72 | 1.00 | 0.97 |
|  |  | |  |  |  |  |  |
| ***LOO in Retraining*** | |  |  |  |  |  |  |
|  | **Sen.** | | **Spec.** | **Acc.** | **AUC** | **PPV*** | **NPV*** |
| *Cosine NN* | 0.75 | | 0.65 | 0.70 | 0.70 | 0.10 | 0.98 |
| *DLDA* | 0.65 | | 0.95 | 0.80 | 0.84 | 0.41 | 0.98 |
| *NSC* | 0.45 | | 0.90 | 0.68 | 0.68 | 0.19 | 0.97 |
| *SVM* | 0.40 | | 0.85 | 0.63 | 0.69 | 0.12 | 0.96 |

***** Performance of models during training, testing, and LOO retraining. Sensitivity, specific, accuracy, and AUC. *PPV and NPV calculated at 5% intracranial aneurysm incidence. Gray shading indicates best performing model. (Acc.=accuracy, AUC=area under the receiver operating characteristic curve, Cosine NN=cosine nearest neighbor, DLDA=diagonal linear discriminant analysis, LOO=Leave-one-out, NSC=nearest shrunken centroids, NPV=negative predictive value, PPV=positive predictive value, Sen.=sensitivity, Spec.=specificity, SVM=support vector machines)
